# Pyroptosis-Related Risk Signature Exhibits Distinct Prognostic, Immune, and Therapeutic Landscapes in Hepatocellular Carcinoma

**DOI:** 10.3389/fgene.2022.823443

**Published:** 2022-03-09

**Authors:** Yidi Zhao, Qingya Song, Fangshi Xu, Yang Zhou, Xiaoli Zuo, Zhengliang Zhang

**Affiliations:** ^1^ Department of Emergency, Second Affiliated Hospital of Xi’an Jiaotong University, Xi’an, China; ^2^ Xi’an Medical Emergency Center, Xi’an, China; ^3^ Department of Medicine, Xi’an Jiaotong University, Xi’an, China; ^4^ Department of Orthopedics, Second Affiliated Hospital of Xi’a Jiaotong University, Xi’an, China

**Keywords:** hepatocellular carcinoma, pyroptosis, risk signature, prognosis, WNK1

## Abstract

**Background:** Hepatocellular carcinoma (HCC) is a common abdominal cancer. The existing therapeutic approaches often fail to achieve satisfactory results. Pyroptosis, an inflammatory form of programmed cell death, provides new ideas for anticancer treatment. However, the roles of pyroptosis-related (PR) genes (PRGs) in HCC remain elusive.

**Methods:** Differentially expressed genes (DEGs) (n = 22) were screened out using TCGA and GTEx databases. A novel PR risk signature was constructed through Lasso regression analysis. Its prognostic value was evaluated through a series of survival analyses and was tested in ICGC and GSE14520 cohorts. CIBERSORT, ssGSEA, and ESTIMATE methods were employed to determine the effects of the PR risk score on the tumor immune microenvironment (TIM). The TIDE scoring system, IMvigor210 cohort, GSE109211 dataset, and GSDC database were applied to explore the associations of the PR risk score with therapeutic effects. The biofunctions of WNK1 in hepatocellular cancer (HC) cells were confirmed through qPCR, colony formation, and Transwell assays.

**Results:** Overall, 22 of 45 PRGs (48.9%) were abnormally expressed in HCC samples. Then, a PR risk signature consisting of eight PRGs was constructed. A high PR risk score led to an unfavorable prognosis. The PR risk score was identified as an independent prognostic factor of HCC and could increase the decision-making benefit of the traditional TNM model. In addition, we established a nomogram containing the clinical stage and PR risk score to predict the survival rates of HCC patients. The prognostic value of the PR model was successfully validated in ICGC and GSE14520 cohorts. Moreover, high PR risk conferred the decreased infiltration level of CD8^+^ T cells and weakened the activities of “cytolytic activity” pathways. As for therapeutic correlation, a high PR risk score seemed to imply a poor efficacy of PD-1/L1 inhibitors and sorafenib. Finally, the overexpression of WNK1 could promote the proliferation, migration, and invasion of HC cells.

**Conclusions:** The PR risk score was closely related to the prognosis, antitumor immune process, therapeutic outcomes, and malignant progression of HCC. WNK1, the core regulator of pyroptosis, possesses pro-oncogenic abilities, showing promise as a novel treatment target.

## Introduction

Hepatocellular carcinoma (HCC) is the fourth common digestive tumor with a non-negligibly high malignancy. In 2021, the estimated new cases of HCC in America reached 42,230, and its cancer-related mortality was as high as 6% (Siegel et al., 2021). Despite consistent improvements in diagnostic and treatment strategies of HCC, the overall survival (OS) is still unsatisfactory. The median survival of HCC patients in China is only 23 months, while that in Japan, the region with the best prognostic outcomes, is less than 60 months (Yang et al., 2019). Hepatic resection is the most effective means for HCC treatment. Unfortunately, more than half of the patients are accompanied by metastatic symptoms at the time of diagnosis (Chen et al., 2020). Although targeted therapy and immune checkpoint inhibitors (ICIs) have been applied in a variety of tumors, including HCC, their efficacy remains limited (Chen et al., 2020). As the first-line systemic therapy for HCC, the median OS of patients who receive either sorafenib or nivolumab is only 10.8 and 13.8 months, respectively (El-Khoueiry et al., 2017; Keating 2017). Moreover, the CheckMate 040 clinical trial has reported that the objective response rate of nivolumab was less than 20% (El-Khoueiry et al., 2017). Hence, finding novel therapeutic strategies and establishing an accurate prognostic system are impending and meaningful. Recently, pyroptosis, an inflammatory form of programmed cell death (PCD), sheds new light on cancer treatment.

Pyroptosis, first proposed by Cookson *et al.* in 2001(Cookson and Brennan 2001), shares the characteristics of necrosis and apoptosis (Ruan et al., 2020). Mechanistically, pyroptosis comprises canonical and non-canonical pathways in which inflammasome, caspase (CASP), and gasdermin (GSDM) serve as the core regulators (Fang et al., 2020; Ruan et al., 2020; Yu et al., 2021). In brief, viruses, toxins, bacteria, dsDNA, chemotherapy drugs, lipopolysaccharide (LPS), and tumor necrosis factor (TNF) activate their corresponding inflammasomes through pattern recognition receptor (PRR) or pathogen-associated molecular pattern (PAMP) modes, which is the initial step of pyroptosis (Xia et al., 2019). Activated inflammasomes mainly consisting of nucleotide-binding oligomerization domain (NOD), toll-like receptor (TLR), and NOD-like receptor (NLR) families disinhibit the CASP family by binding to apoptosis-associated speck-like proteins (ASCs). Subsequently, the CASP family, especially CASP1/4/5, cleaves GSDM proteins to expose their functional N-terminal domain, which thereby induces pore-independent cell lysis and releases inflammatory mediators, such as IL-1β and IL-18 (Ruan et al., 2020).

Abundant evidence has reiterated that pyroptosis is closely involved in cancer progression, drug resistance, and the antitumor immune process (Fang et al., 2020). It is conceivable that pyroptosis has great potential to treat cancer. For example, BRAF and MEK inhibitors can regulate the tumor immune microenvironment (TIM) through the pyroptosis process (Erkes et al., 2020). Hydrogen inhibits endometrial cancer progression by activating the CASP1/GSDMD-mediated pyroptosis pathway (Yang et al., 2020). To date, some studies have explored the roles of pyroptosis regulatory genes (PRGs) in endometrial cancer (Zhang and Yang 2021), ovarian cancer (Ye et al., 2021), and breast cancer (Lv et al., 2021). These findings undoubtedly point toward the fact that pyroptosis profoundly affects the prognosis and immune process in multiple cancers. Although there is also some research reporting that pyroptosis-related (PR) genes have crucial functions in HCC (Liu, Shao, Bu, Xu, and Shi 2021; Zheng et al., 2021), its underlying mechanisms remain incompletely understood. In view of this, we constructed a novel PR risk signature through Lasso regression analysis based on an improved PR gene set. Its prognostic value, immune effect, and therapeutic correlation were comprehensively elucidated. Furthermore, the biofunctions of WNK1 that was the core component of the PR model were also determined through *in vitro* cell experiments. Our findings provide new evidence for applying pyroptosis in the treatment of HCC.

## Materials and Methods

### Data Source

The clinical information and gene expression data were derived from TCGA (https://portal.gdc.cancer.gov/), ICGC (https://dcc.icgc.org/releases), and GEO (https://www.ncbi.nlm.nih.gov/geo/) databases. In total, 110 normal liver tissue samples obtained from the GTEx database (https://xenabrowser.net/datapages/) were applied to offset the shortage of TCGA normal samples (n = 50), and 28 TCGA samples were excluded owing to their too short follow-up (less than 30 days). A Japanese project named ICGC-LIRI (n = 221) and GSE14520 (n = 221) were used as validation cohorts (Sun et al., 2020; Xie et al., 2021). Transcriptome data were standardized by log2 (FPKM+1) transformation. The clinical characteristics of TCGA, ICGC, and GEO cohorts are presented in Table 1.

**TABLE 1:** Clinical characteristics of TCGA, ICGC, and GSE14520 cohorts.

Item	TCGA	ICGC	GSE14520
Sample size	377	231	221
Survival status			
Dead	128	42	85
Alive	249	189	136
Age (years)			
<60	172	44	178
≥60	204	187	43
Unknown	1	0	0
Histological grade		NA	NA
G1	55	—	—
G2	180	—	—
G3	124	—	—
G4	13	—	—
Unknown	5	—	—
Clinical stage			
I	175	36	93
II	87	105	77
III	86	71	49
IV	5	19	0
Unknown	24	0	2
T		NA	NA
T1	185	—	—
T2	95	—	—
T3	81	—	—
T4	13	—	—
Unknown	3	—	—
M		NA	NA
M0	272	—	—
M1	4	—	—
Unknown	101	—	—
N		NA	NA
N0	257	—	—
N1	4	—	—
Unknown	116	—	—

NA, not available.

### Improved Pyroptosis-Related Gene Set

In the present study, we established an improved PR gene set which composed of four major segments. First, several researchers used the same PR gene set containing 33 members to probe into the roles of PRGs in cancers (Shen et al., 2021; Lin et al., 2021; Ye et al., 2021). These PRGs were also adopted in our study. Second, we incorporated a PR gene set (n = 27) from the Molecular Signatures Database (MSigDB) (https://www.gsea-msigdb.org/gsea/msigdb/) into our PR gene set. Third, as recently mentioned by Ruan et al. (2020) and Fang et al. (2020), there is a bypass pathway responsible for regulating pyroptosis, in which TLR3, TLR4, RIPK1, DIABLO (Smac), CASP8, and GSDMD participate in. Fourth, several new findings expand pyroptosis regulatory members (Linder et al., 2020; Dong et al., 2021; Mayes-Hopfinger et al., 2021). CARD8 has been identified as a new inflammasome to trigger pyroptosis (Linder et al., 2020). WNK1 can decelerate NLRP3 activation by balancing intracellular Cl- and K+ concentrations (Mayes-Hopfinger et al., 2021). BRD4 has the ability to regulate NLRC4 inflammasome activation by facilitating the transcription of Naips (Dong et al., 2021). Finally, we established an improved PR gene set consisting of 45 members (Supplementary Table S1).

### Construction of Pyroptosis-Related Risk Signature

The PR differentially expressed genes (DEGs) were screened out by the “Limma” package in R software (ver 3.6.3). The threshold for screening criterion was set at an adjusted *p*-value<0.05 and the absolute value of Log_2_FC ≥ 0.58 (1.5-fold difference in gene expression). Next, PR DEGs were entered into Lasso regression analysis to construct a novel PR risk signature in HCC.

### Assessments of Prognostic Value

The Cutoff Finder online tool (http://molpath.charite.de/cutoff) was used to determine the optimal cutoff value of the PR risk score. According to the value, patients were divided into high- and low-risk groups, and then their survival differences were performed based on the Kaplan–Meier method. Cox univariate and multivariate analyses were employed to determine the independent prognostic factors. The predictive accuracy of the PR risk signature was evaluated by the receiver operating characteristic curve (ROC). Decision curve analysis (DCA) was applied to assess the gain effects of the PR risk score on two traditional prognostic models of HCC. Traditional model A consisted of age, histological grade, and clinical stage based on a multivariate logistic regression algorithm. Traditional model B consisted of age, histological grade, and TNM staging. Except for the M1 stage (n = 4), we performed clinical subgroups for clinically important variables. A nomogram was constructed to predict the OSR of individuals at 1, 3, and 5 years. In addition, the ICGC-LIRI and GSE14520 datasets served as validation cohorts.

### Immune Analyses

The CIBERSORT algorithm was applied to calculate the immune abundances of 22 lymphocyte subtypes in each HCC sample. The activities of 13 immune-related pathways were quantified based on the single-sample gene set enrichment analysis (ssGSEA) method. The ESTIMATE algorithm is capable of inferring the immune cell admixture in tumor parenchyma and stroma, thereby depicting the tumor immune microenvironment (TIM) (Luo et al., 2020; Meng et al., 2020). The TIMER web server is a comprehensive resource for systematical analysis of immune infiltrates across diverse cancer types (Li et al., 2017). The correlations between the infiltrating levels of immune cells and the expressions of hub PRGs were confirmed using the TIMER database.

### Therapeutic Correlation Analyses

We investigated the potential linkages between the PR risk score and therapeutic effects from three perspectives. First, we analyzed the efficacy of programmed death 1/ligand 1 (PD-1/L1) inhibitor. Given that the expression status of immune checkpoints (ICs) profoundly affects the tumor response to ICIs (Brueckl et al., 2020; Sivapiragasam et al., 2021), we analyzed the expressive correlations between six pivotal ICs and the PR risk score based on the Spearman algorithm. The expressive differences of ICs between different PR risk levels were also compared. Of note, Jiang P et al. have developed TIDE scores to predict patients’ response to anti-PD-1/L1 and anti-CTLA4 treatments based on computational estimation of T-cell dysfunction and tumor immune evasion (Jiang et al., 2018). Therefore, we calculated the TIDE scores in high- and low-PR risk groups. Furthermore, a real clinical cohort, IMvigor210 (Balar et al., 2017), was used to reconfirm the differences in the PR risk score between different therapeutic outcomes of the PD-1 inhibitor, atezolizumab. Second, the efficacy of sorafenib was analyzed. Since sorafenib is the first-line option for HCC systemic treatment, we applied the GSE109211 dataset (Pinyol et al., 2019) to compare the differences in the PR risk score between response and non-response patients. The GSE109211 dataset was derived from the phase 3 STORM trial, containing 140 HCC patients receiving sorafenib adjuvant treatment. Third, the effects on drug sensitivity were analyzed. The Genomics of Drug Sensitivity in Cancer (GDSC) database (https://www.cancerrxgene.org/) (Yang et al., 2013) provided vast information on drug sensitivity in cancer cells and molecular markers of drug response. Using the GDSC database, we ascertained the relationships between the expressions of crucial PRGs and the sensitivities (IC50) of some classical chemotherapeutic and molecular targeted (MT) drugs. The clinical information of IMvigor210 and GSE109211 cohorts is presented in Supplementary Table S2.

### HPA Database

The Human Protein Atlas (HPA) database exhibited the landscape of cancer proteomes in 32 different tissues and organs (https://www.proteinatlas.org/) (Uhlén et al., 2015). Through the immunohistochemistry images in the HPA database, we compared the differences in protein expression levels of crucial PRGs between normal liver and HCC tissues.

### Cell Culture and Transfection

Two human hepatocellular cancer cell lines (HepG2 and Huh-7) and one normal liver cell line (THLE-3) were purchased from Fenghui Biotechnology Company (Hunan, China). HepG2 and THLE-3 cells were both cultured in Minimum Essential Medium (MEM) containing 10% fetal bovine serum (FBS) and 1% penicillin/streptomycin (P/S) (Procell, Wuhan, China). Huh-7 cells were cultured in Dulbecco’s modified Eagle medium (DMEM) containing 10% FBS and 1% P/S (Procell, Wuhan, China). Specific shRNA and amplification plasmids were designed by HanHeng Biotechnology (Shanghai, China). The detailed sequences of sh-WNK1 and OE-WNK1 are shown in Supplementary Table S3. Lentiviruses (HanHeng Biotechnology, Shanghai, China) were responsible for transfecting liver cancer (LC) cells.

### RT-qPCR

Total RNA was extracted by using TRIzol reagent (TakaRa, Japan). RNA concentration was calculated by using the A260/A280 ratio (Nanodrop 2000 spectrophotometer). cDNA was synthesized using the PrimeScript RT reagent kit (TaKaRa, Japan). RT-qPCR was tracked by using SYBR-Green PCR reagent (Takara, Japan) and performed on the ABI Prism 7900 sequence detection system. GAPDH was used as an internal reference. RNA relative expression was calculated based on the 2^−ΔΔCT^ method. The WNK1 forward primer sequence was 5′- GAC​TCC​CGG​CGC​CAT​TTA​G-3′; the WNK1 reverse primer sequence was 5′- AGA​GAA​AAG​AGC​AGC​CAC​CC-3; the GAPDH forward primer sequence was 5′-GTC​GCC​AGC​CGA​GCC​ACA​TC-3; and the GAPDH reverse primer sequence was 5′-CCA​GGC​GCC​CAA​TAC​GAC​CA-3'.

### Colony Formation Assay

After 48-h transfection, cells at the log phase were made into single-cell suspensions by trypsinization. Then, the transfected cells were seeded into 6-well plates at a density of 1×10^3^/per well. When visible clones appeared (about 2–3 weeks), the incubation was terminated. Macroscopic colonies were fixed by methanol and stained by Giemsa stain. Cell colonies were counted under a microscope from five random fields.

### Transwell Migration and Invasion Assays

Transwell migration and invasion assays were performed as described in a previous study (Xu et al., 2021). For this assay, 100 μL cell suspensions (2 × 10^4^ per well) were placed into 24-well Transwell chambers (Corning, NY, United States). DMEM or MEM containing 0.1% FBS was added to upper chambers, while that with 10% FBS was added to lower ones. After 48-h incubation, the migratory cells were fixed by paraformaldehyde and stained with 0.1% crystal violet. Cell counting was performed under a high-magnification microscope (100-fold) from five random visual fields. For invasion assays, the upper chambers were pre-coated with Matrigel.

### Statistical Analysis

All statistical analyses were conducted by using R software (version 3.6.2) and GraphPad Prism (version 8.0.1). The Kruskal–Wallis test was used to compare statistical differences between multiple groups. The Wilcoxon rank-sum test was applied for the analysis of categorical variables. A *p*-value < 0.05 was considered statistically significant.

## Results

### A Novel PR Risk Signature Is Composed of Seven Pivotal Pyroptosis Regulators

**FIGURE 1:**
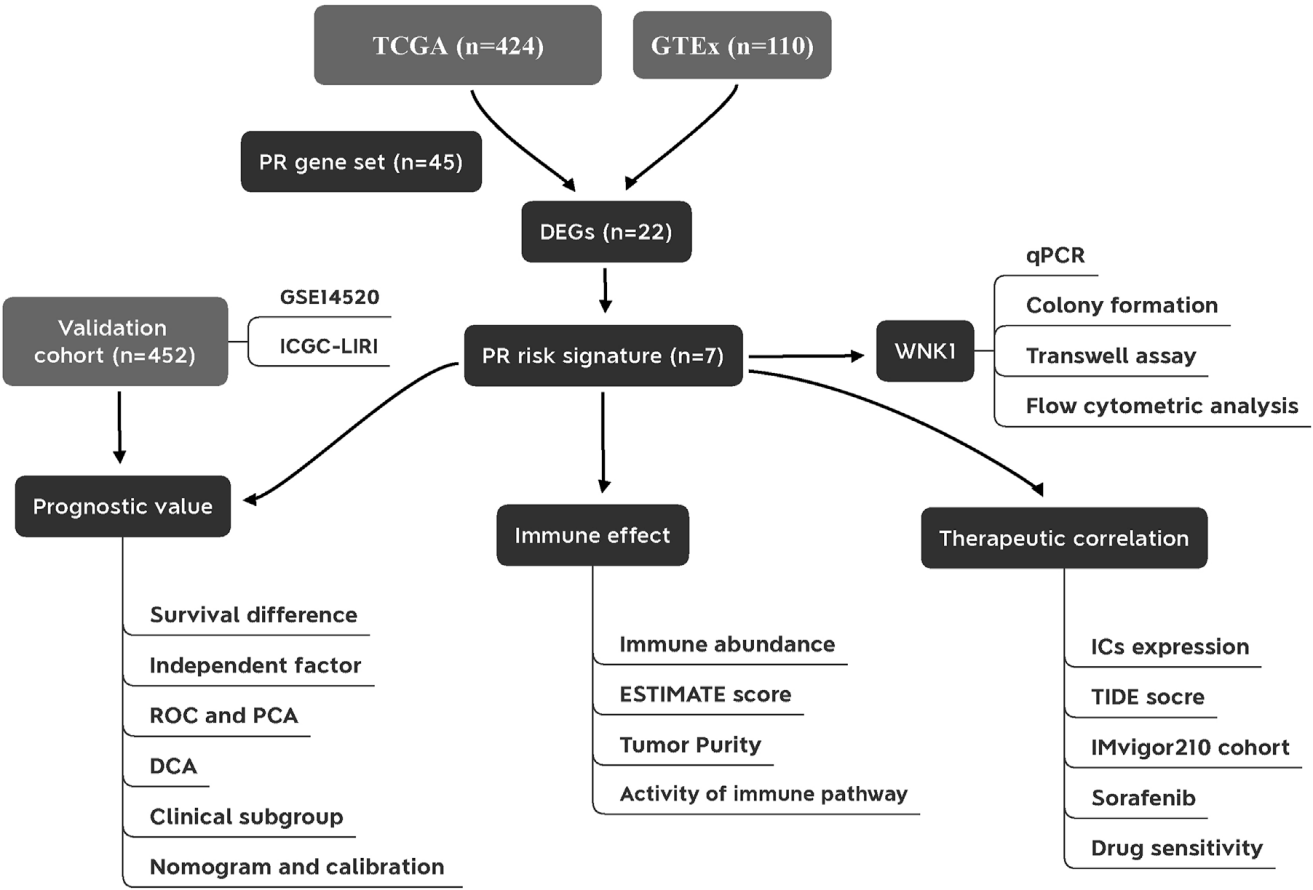
Flowchart of the present study. PR, pyroptosis-related; DEGs, differentially expressed genes; ROC, receiver operating characteristic curve; PCA, principal component analysis; DCA, decision curve analysis; ICs, immune checkpoints.

**FIGURE 2:**
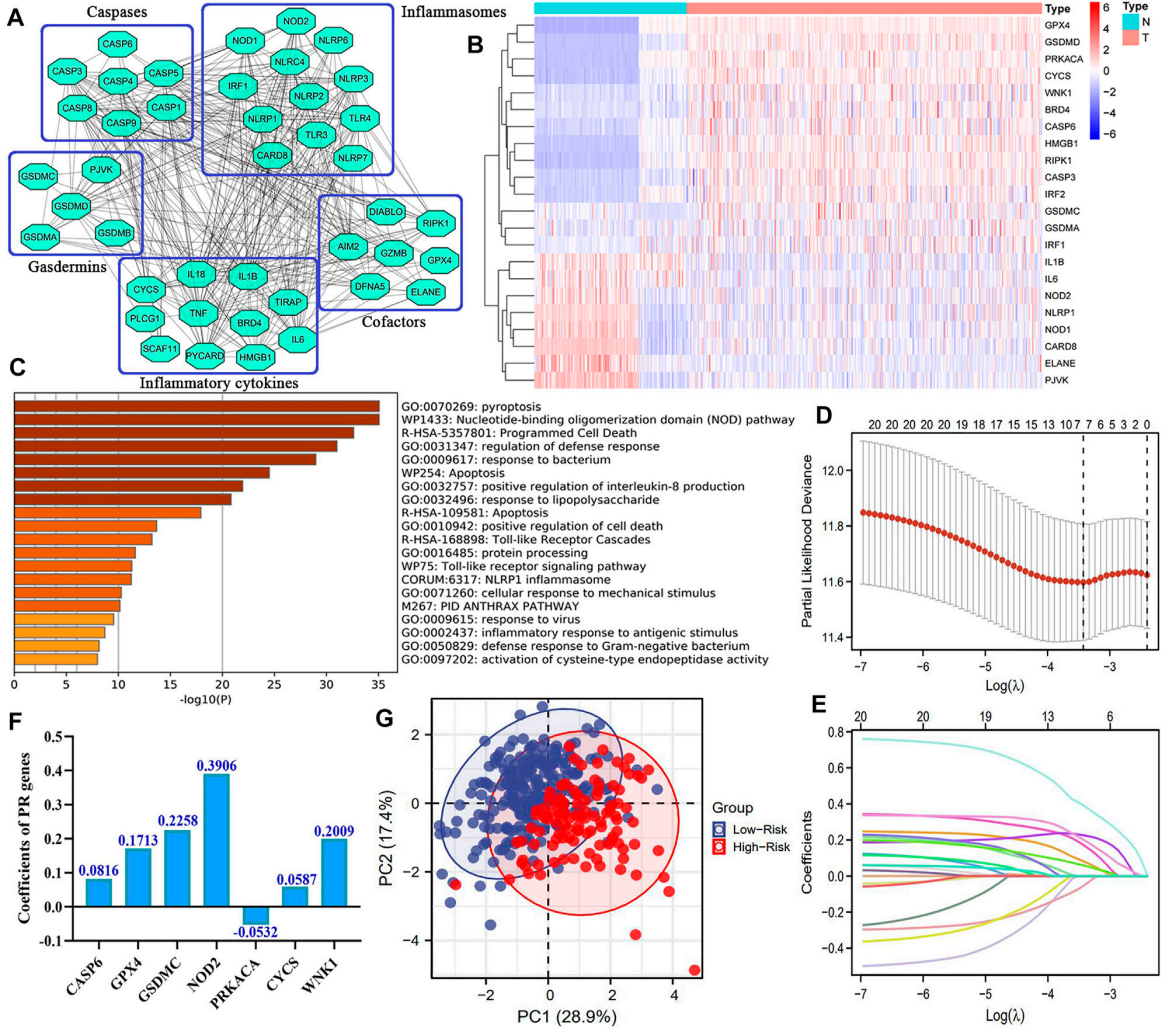
Novel PR risk signature. **(A)** The PPI network of 45 core pyroptosis regulatory genes. **(B)** The heatmap of pyroptosis DEGs. **(C)** The biological functions of 45 PRGs **(D–E)** The process of Lasso regression analysis **(F)** The coefficients of each PRG in PR risk signature. **(G)** The PCA plots. PR, pyroptosis-related; PPI, protein–protein interaction; DEGs, differentially expressed genes; PRGs, pyroptosis-related genes; PCA, principal component analysis.

The flowchart of this study is presented in Figure 1. We first constructed a comprehensive and reliable PR gene set. As mentioned in Introduction, PRR/PAMP—inflammasome interaction drives pyroptosis initiation. Next, activated inflammasomes convert the precursors of CASPs to their functional status. In the effector phase, cleaved GSDM members trigger cell lysis, thus releasing intracellular inflammatory cytokines. These point toward the fact that inflammasomes, caspases, and gasdermins act as three core links of pyroptosis. The protein–protein interaction (PPI) network of 45 key PR regulators is shown in Figure 2A. Biological functional analyses indicated that these genes were enriched in “pyroptosis,” “programmed cell death,” “apoptosis,” etc*.* pathways (Figure 2C), which solidly proved the plausibility of our PR gene set. This laid the foundation for modeling.

Overall, 22 of 45 PRGs (48.9%) were abnormally expressed in HCC samples. Among them, eight PRGs (ELANE, PVJK, IL6, IL1β, CARD8, NOD2, NOD1, and NLRP1) were downregulated, while the rest were upregulated (CASP6, GPX4, GSDMC, CYCS, WNK1, etc*.*) (Figure 2B). These PR DEGs were selected to enter the Lasso regression analysis, by which a novel PR risk signature was constructed (Figure 2D–E). The PR risk model was as follows (Figure 2F): the PR risk score = 0.0816*(CASP6 relative expression) + 0.1713*(GPX4 relative expression) + 0.2258*(GSDMC relative expression) + 0.3906*(NOD2 relative expression) + (-0.0532)*(PRKACA relative expression) + 0.0587*(PLCG1 relative expression) + 0.2009*(WNK1 relative expression). PCA analysis revealed that our PR risk model possessed an acceptable model fit, which could explain 46.3% of the total prognostic variation (Figure 2G).

### PR Risk Signature Serves as an Important Supplement to the Prognostic Assessment of HCC

**FIGURE 3:**
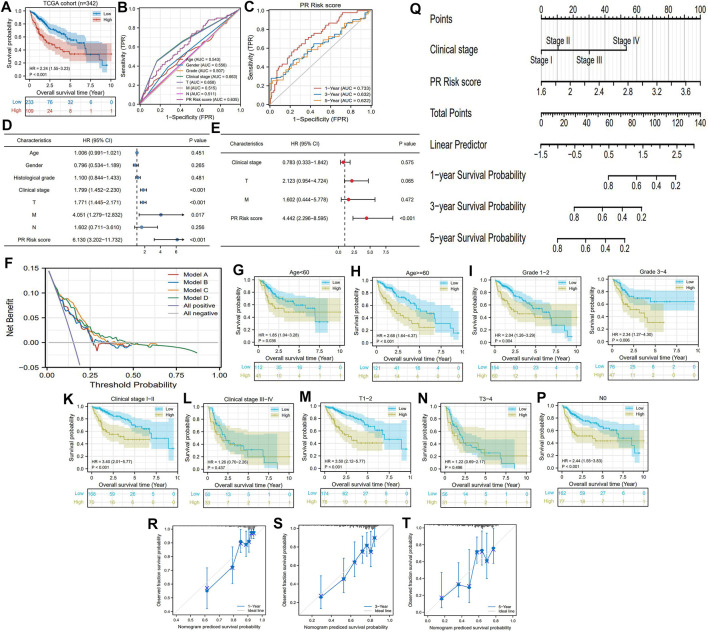
Prognostic value of PR risk signature in HCC **(A)** The OS difference between high- and low-PR risk groups. **(B)** ROC curves of clinicopathologic features and PR risk score. **(C)** Time-dependent ROC curves of PR risk score. **(D)** The univariate independent prognostic analysis. **(E)** The multivariate independent prognostic analysis. **(F)** DCA results. “Model A” is the traditional prognostic model composed of age, histological grade, and clinical stage. “Model B” is the traditional prognostic model composed of age, histological grade, and TNM staging. “Model C and D” are the improved models A and B, respectively, with the PR risk score added. **(G–P)** The OS difference between high- and low-PR risk groups in each clinical subgroup **(Q)** The nomogram is used for predicting 1-, 3-, and 5-year overall survival probability of HCC patients **(R–T)** The calibration curves. PR, pyroptosis-related; HCC, hepatocellular carcinoma; OS, overall survival; ROC, receiver operating characteristic curve; DCA, decision curve analysis; HR, hazard ratio.

According to the optimal cutoff value of the PR risk score (2.438), 342 HCC patients in the TCGA cohort were categorized into different risk groups. Patients with high risk suffered a poor survival outcome, whose 5-year OSR was less than 35% (Figure 3A). Meanwhile, the PR risk score had a moderate predictive performance (AUC = 0.635, Figure 3B), which trumped age, gender, histological grade, and M and N stages but was slightly weaker than clinical and T stages. Its predictive ability decreased gradually with the prolonging time and was highest for predicting 1-year OSR (AUC = 0.733, Figure 3C). Univariate and multivariate regression analyses indicated that clinical stage, TM stages, and PR risk score were all related to prognosis, but only the PR risk score was identified as an independent prognostic factor of HCC (HR = 4.442, P<0.001) (Figure 3D–E). It is worth noting that introducing the PR risk score into traditional prognostic models (models A and B) could boost their decision-making benefit (models C and D) (Figure 3F).

The PR risk score was also applicable for different subgroups of patients. It could distinguish the prognostic differences of HCC patients with age<60, age ≥60, grade 1/2, grade 3/4, clinical stage I/II, T1/2, M0, and N0 (Figure 3G–K, M, O-P), whereas failed in middle and advanced patients (Figure 3L, N). From the perspective of clinical practice, we established a nomogram containing clinical stage and PR risk score to predict the survival rates of HCC patients (Figure 3Q). For instance, a clinical stage II patient with a PR risk score of 2 would obtain a predicted 3-year OSR of 80%, while a patient with a PR risk score of 2.8 would obtain that of 60%. In addition, the calibration plots indicated that the predicted probabilities matched well with actual survival rates (Figure 3R–T). Collectively, PR risk signature contributed indispensable information for prognostic assessment of HCC patients.

Moreover, we also analyzed the relationships between the PR risk score and clinicopathologic features of HCC. As shown in Supplementary Figure S1A, PR risk scores in advanced clinical stages were significantly higher than those in localized ones, which indicated that PR genes may participate in the progression of HCC. It is currently known that Lynch syndrome is the genetic carcinogenesis cause of multiple cancers. Casper M *et al.* have found that HCC is an extracolonic manifestation of Lynch syndrome, and SEC63 may serve as a potential biomarker for this hepatocarcinogenesis (Casper et al., 2013). In the current study, we observed that SEC63 expression was positively correlated with the PR risk score (Supplementary Figure S1B), suggesting that high PR risk contributed to HCC onset.

### The Prognostic Value of PR Model Is Successfully Validated in External Cohorts

**FIGURE 4:**
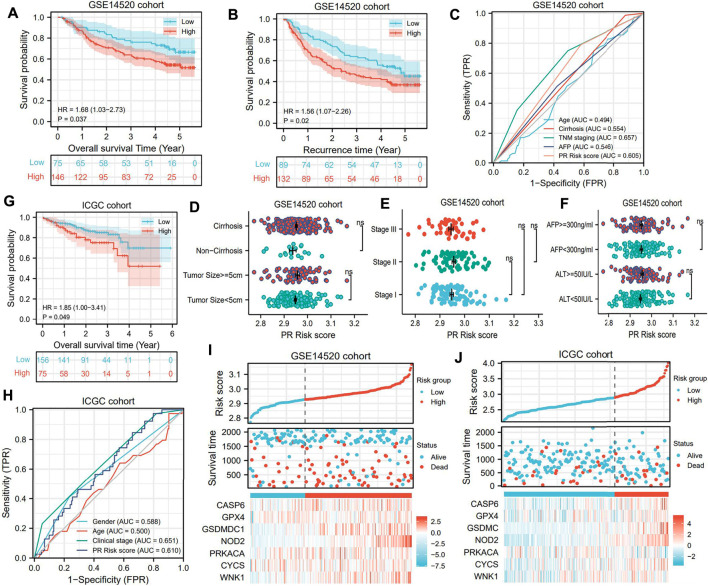
PR risk signature is successfully validated in two external cohorts. **(A)** The OS difference in GSE14520 cohort. **(B)** The RFS difference in GSE14520 cohort. **(C)** The predictive accuracy of PR model in GSE14520 cohort. **(D–F)** The relationships between PR risk levels and HCC clinical parameters in GSE14520 cohort. **(G)** The OS difference in ICGC cohort. **(H)** The predictive accuracy of PR model in ICGC cohort. **(I–J)** The risk plots of two validation cohorts. PR, pyroptosis-related; OS, overall survival; RFS, recurrence-free survival; HCC, hepatocellular carcinoma.

GSE14520 and ICGC datasets were utilized as validation cohorts to test the prognostic value of the PR model. Significant differences were observed between high- and low-risk groups in terms of either overall survival time or recurrence time (Figure 4A,B). Analogous to the TCGA cohort, the PR risk score still presented a moderate predictive capacity (AUC = 0.605, Figure 4C). Nevertheless, the PR risk score was not correlated with tumor size, history of cirrhosis, clinical stage, and serum levels of AFP and ALT (Figure 4D-F). In the ICGC cohort, high PR risk also rendered an unfavorable prognosis (Figure 4G), and its prediction accuracy was 0.610 (Figure 4H). These results pointed to the same conclusion, which was PR risk model was widely applicable. The risk plots of GSE14520 and ICGC cohorts are exhibited in Figure 4I,J.

### PR Risk Is Closely Associated With Tumor Immune Process of HCC

**FIGURE 5:**
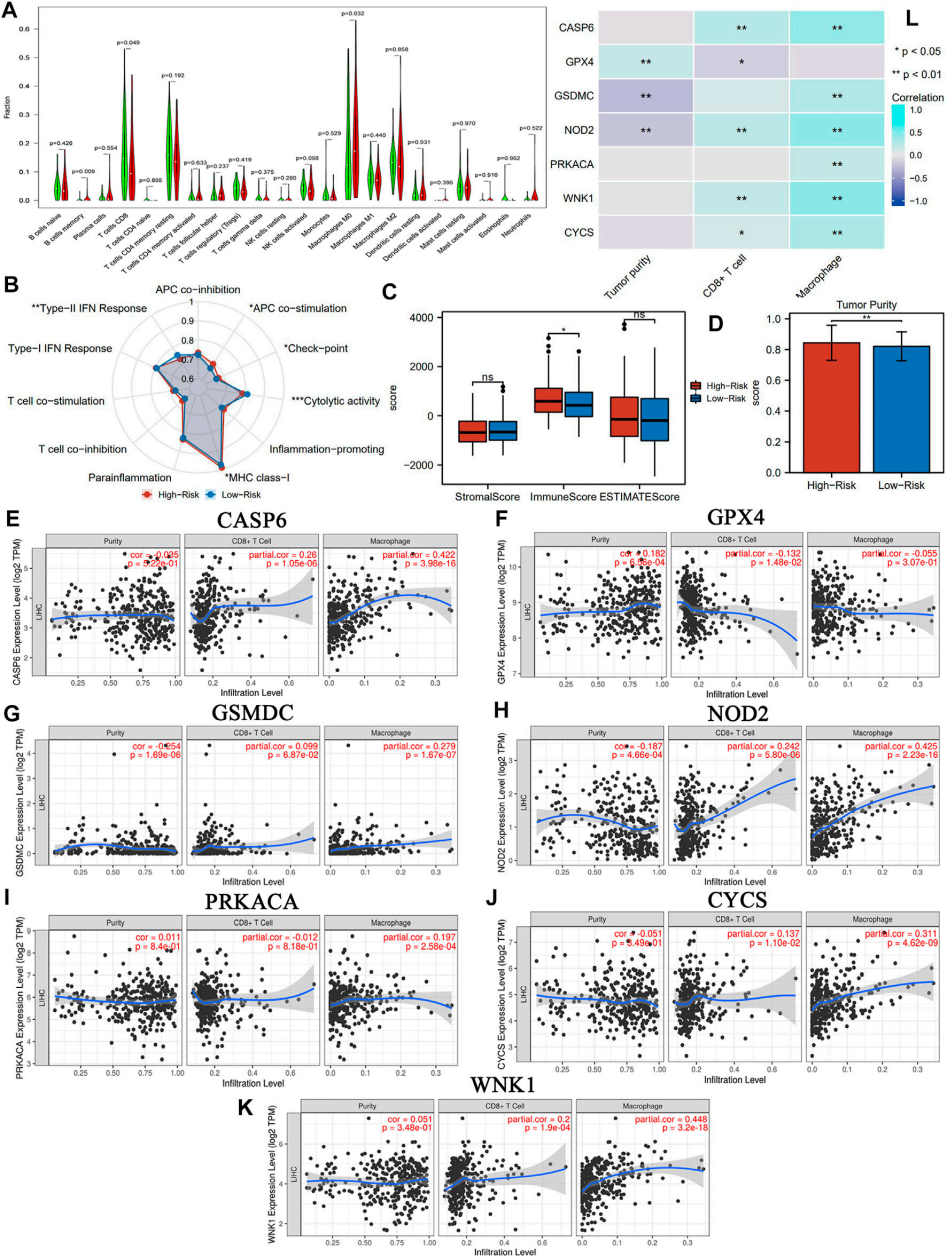
PR risk score is closely associated with TIM **(A)** The distribution of the immune abundance of 22 leukocyte subtypes in each HCC sample. **(B)** The effects of PR risk level on the activities of 11 immune-related signaling pathways. **(C)** The differences in the stromal score, immune score, and ESTIMATE score. **(D)** The differences in tumor purity. **(E–K)** The correlations between PR risk score, and the infiltration levels of CD8^+^ T cells and macrophages based on TIMER database. **(L)** The summary of immune correlations. PR, pyroptosis-related; TIM, tumor immune microenvironment; HCC, hepatocellular carcinoma; *p < 0.05, **p < 0.01.

The PR risk score profoundly reflected the tumor immune microenvironment (TIM) of HCC. The immune abundances of 22 leukocyte subtypes were variable in each HCC sample (Supplementary Figure S2). The infiltration level of CD8^+^ T cells was significantly reduced in the high-PR risk group compared with that in the low-PR risk group (Figure 5A). Inversely, the immune enrichments of macrophages M0 and B cells memory were markedly decreased in the high-PR risk group (Figure 5A). Moreover, the PR risk score was tightly associated with the activities of immune signaling pathways. The activities of “APC co-stimulation,” “checkpoint,” and “MHC class I” pathways were enhanced, whereas that of “cytolytic activity” and “type-II IFN response” pathways were markedly restrained in the high-PR risk group (Figure 5B). Consulting some crucial immunological research (Cassetta and Pollard 2018; Castro et al., 2018; Farhood et al., 2019; Cornel et al., 2020; Sadeghzadeh et al., 2020; Stocker et al., 2020; Wculek et al., 2020), we found that the affected immune cells and immune signaling pathways have been proven to intimately participate in the regulation of antitumor immunity and the immune tolerance (Table 2). These findings manifested that the PR risk score had a tight and complex linkage to the TIM of HCC. The immune heatmaps of different PR risk levels are exhibited in Supplementary Figure S3.

**TABLE 2:** Associations between PR risk score and TIM.

Immune cell and pathway	Variation trend in high-PR risk	Roles in tumor immunity	Final effect on antitumor immune
T cells CD8	Decreased	CD8^+^ T cells possess potent abilities to eradicate tumor cells and can induce the immunosensitivity of cancer cells	Unfavorable
Macrophages M0	Increased	Aggregation of macrophages in solid tumors commonly leads to poor prognosis and chemotherapy resistance through promoting genetic instability, nurturing cancer stem cells, and supporting metastasis	Unfavorable
APC co-stimulation	Increased	Double-edged sword: On the one hand, APCs strengthen CD8^+^ T cells immunity through cross-presentation of antigens; on the other hand, they drive immune tolerance by limiting co-stimulatory signaling of T cells	Uncertain
Type-II IFN Response	Increased	Dual roles: Type-II IFN commonly has antiproliferative, pro-apoptotic, and antitumor competencies. Conversely, it could play a protumorigenic role through IFN-γ signaling insensitivity	Uncertain
MHC class-I	Increased	MHC-I is core site for CD8^+^ T cells to recognize tumor cells	Favorable
Check-point	Increased	Expressions of ICs on tumor cells contribute to immune evasion	Unfavorable
Cytolytic activity	Decreased	CD8^+^ T cells, NK cells, and CTLs act as main effector cells in antitumor immunity	Unfavorable

PR, pyroptosis-related; TIM, tumor immune microenvironment; APCs, antigen-presenting cells; IFN, interferon; MHC, major histocompatibility complex; ICIs, immune checkpoints; CTLs, cytotoxic T lymphocytes.

As for the immune-related score, no significant differences were observed in stromal and ESTIMATE scores between high- and low-risk groups (Figure 5C). Nevertheless, both immune score and tumor purity in the high-risk group were notably higher than those in the low-risk group (Figures 5C,D). In addition, the expression level of seven pivotal PRGs exhibited a certain correlation with the tumor purity and the infiltration level of CD8^+^ T cells and macrophages (Figures 5E–K). Interestingly, the expressions of some PRGs were positively correlated with the immune abundance of CD8^+^ T cells (Figure 5L). However, these positive correlations may imply the establishment of immune resistance and tolerance. Take WNK1 as an example, the increased expression of WNK1 only promoted the enrichment of CD8^+^ T cells but could not decrease tumor purity. It may suggest that increasing CD8^+^ T cells could not penetrate the immune barrier and accumulated only in the tumor stroma, rather than parenchyma.

### PR Risk Score May Hint the Therapeutic Effects of PD-1/L1 Inhibitor and Sorafenib

**FIGURE 6:**
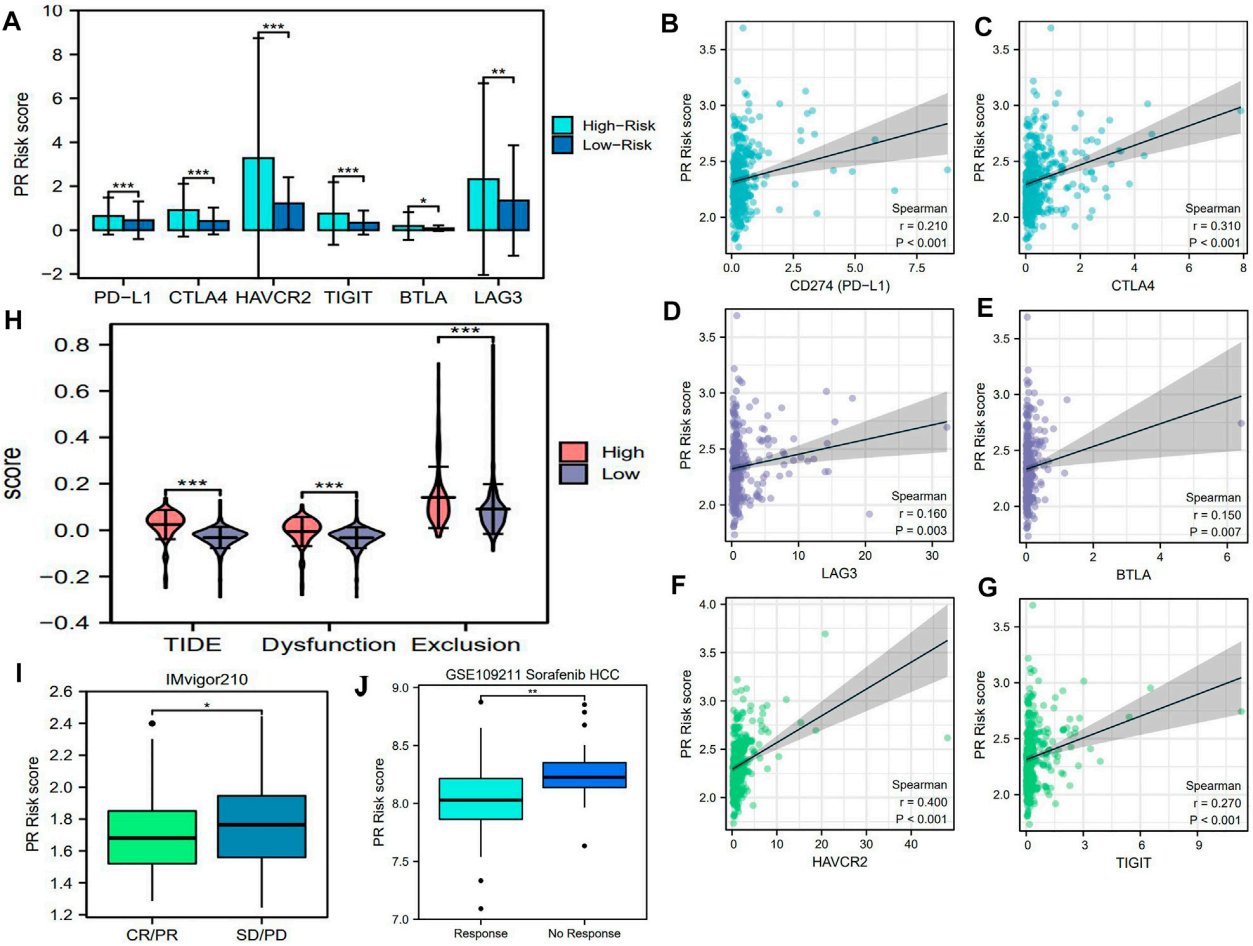
PR risk score implies the efficacy of PD-1/L1 inhibitors and sorafenib. **(A)** The expression differences of six pivotal ICs between high- and low-PR risk. **(B–G)** The correlations between PR risk score and the expressions of six ICs. **(I)** The differences in PR risk score between therapeutic response and non-response patients in IMvigor 210 cohort. **(J)** The differences in PR risk score between sorafenib response and non-response patients in GSE109211 cohort. PR, pyroptosis-related; PD-1/L1, programmed death 1/ligand 1; ICs, immune checkpoints; *p < 0.05, **p < 0.01, ***p < 0.001.

As an emerging therapeutic approach, PD-1/L1 inhibitors bring a new approach for HCC treatment. Nivolumab (PD-1 inhibitor), pembrolizumab (PD-1 inhibitor), and atezolizumab (PD-L1 inhibitor) have been recommended as the common means for HCC systemic treatment by the latest National Comprehensive Cancer Network (NCCN) guideline (Benson et al., 2021). Although the biomarkers for predicting the efficacy of ICIs were still inconclusive, patients with the overexpression of immune checkpoints (ICs) usually presented a good response to this intervention (Patel and Kurzrock 2015; Amrane et al., 2020). Therefore, we investigated the expressive correlations between six pivotal ICs and PR risk score. As shown in Figure 6A, the expressions of six ICs in the high-risk group were higher than those in the low-risk one. Besides, IC expressions were all positively correlated with the PR risk score (Figures 6B–G). These findings seem to indicate that high PR risk was conducive to ICI treatment.

Nevertheless, further analyses contradicted the aforementioned assumption. First, the TIDE score and the score of immune exclusion in the high-risk group were both notably higher than those in the low-risk group (Figure 6H), which heralded that patients with high PR risk scores had a greater tendency to suffer worse therapeutic effects (Jiang et al., 2018). Second, according to a real clinical cohort IMvigor210 (Balar et al., 2017), the PR risk score of the disease progression cases was still higher than that of atezolizumab response cases (Figure 6I). These findings manifested that high PR risk was detrimental to ICI treatment.

**FIGURE 7:**
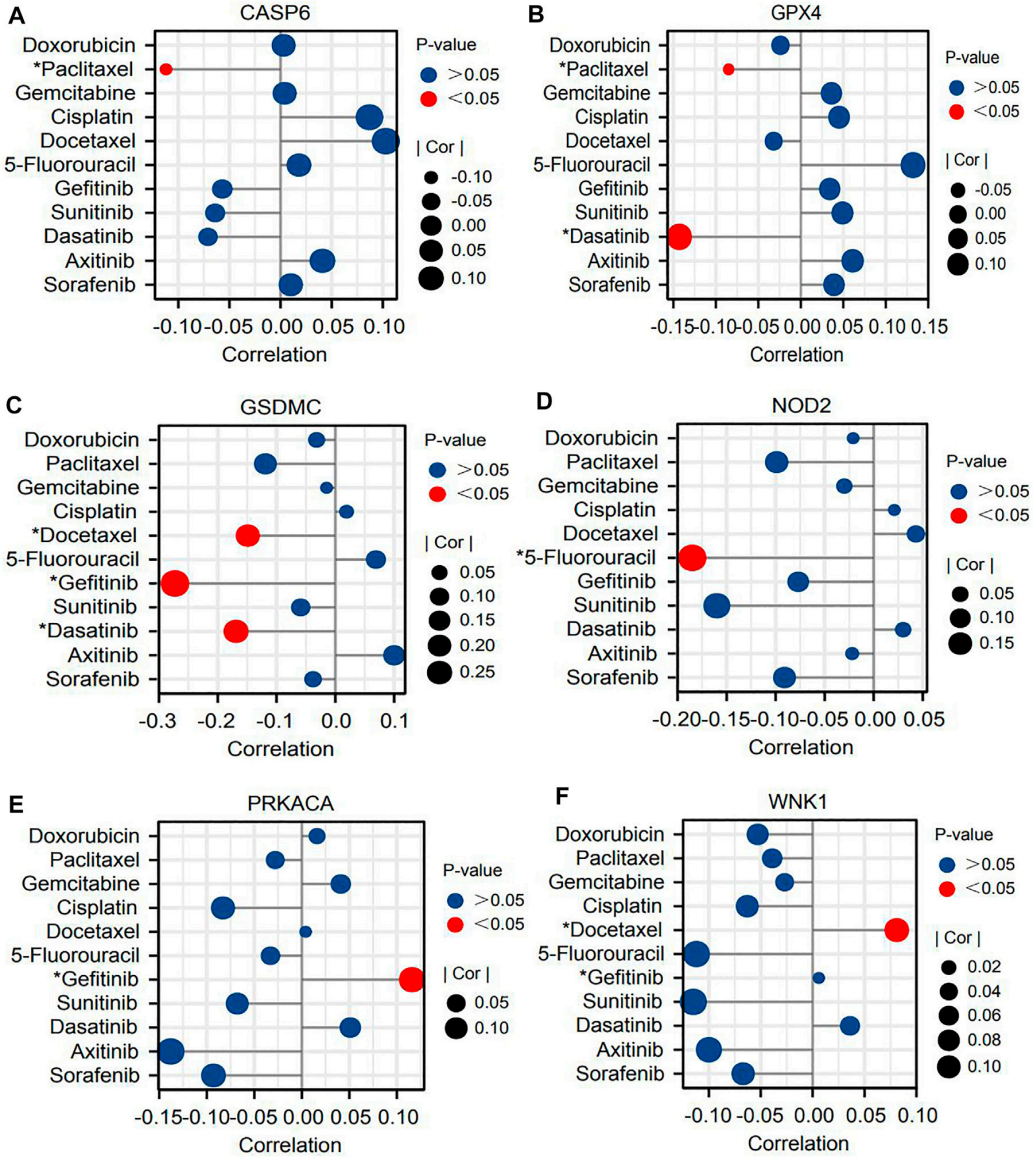
Six core PRGs may affect the sensitivities of multiple molecular target drugs and chemotherapy drugs based on the GSDC database. **(A–F)** The correlations between the expressions of six PRGs and IC50 values of eleven drugs. PRGs, pyroptosis-related genes; IC50, half-maximal inhibitory concentration; *p < 0.05.

Next, we probed the relationships between core PRGs and the sensitivities of multiple classical chemotherapies and molecular targeted drugs. As the first-line option against HCC, sorafenib, its efficacy was found to have a certain association with PR risk score. The PR risk score of patients with response to sorafenib was significantly higher than that of patients with no response to sorafenib (Figure 6J). Unexpectedly, six PRG expressions could not affect its sensitivity based on the GSDC database (Figures 7A–F). Moreover, the expressions of CASP6, GPX4, GSDMC, and NOD2 could increase the susceptibility of tumor cells to paclitaxel, 5-fluorouracil, dasatinib, and gefitinib (Figures 7A–D). However, the expressions of PRKACA and WNK1 decreased the susceptibility to gefitinib and docetaxel (Figures 7E,F).

### PRGs Differentially Express in Liver Cancer Tissues

**FIGURE 8:**
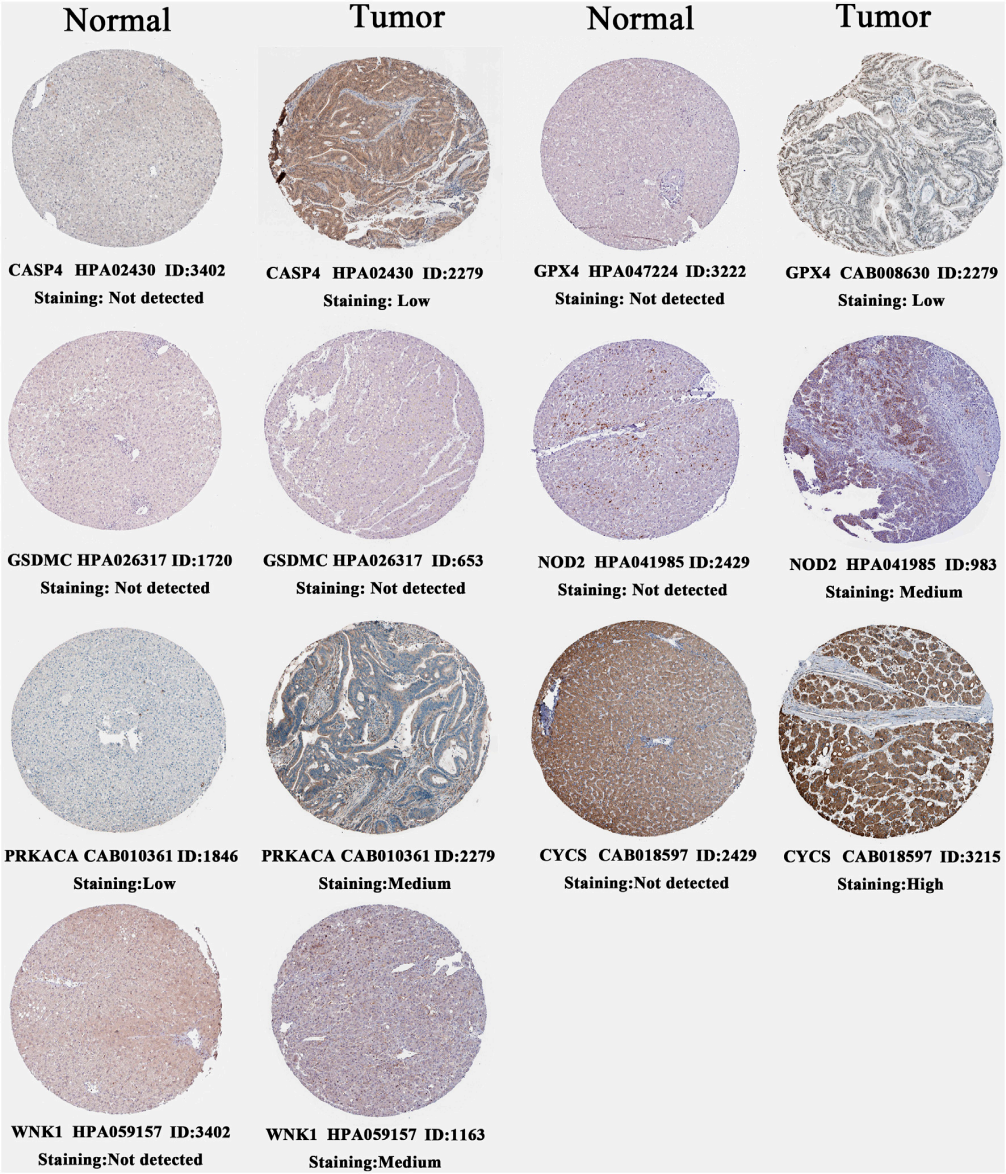
Histological expressions of seven core PRGs based on the HPA database. The top of the figure indicates the category of the tissue specimen. The name of PRG, antibody type, patient ID, and staining intensity are listed at the bottom of each image. PRGs, pyroptosis-related genes.

We confirmed the histological expressions of seven pivotal PRGs through the HPA database (Figure 8). In normal liver tissue, all model genes were barely detectable at the histological level. Compared with normal tissues, CASP6, GPX4, NOD2, and WNK1 were upregulated in HCC samples, presenting low- to medium-intensity expressions, and high expression of CYCS emerged in tumor tissues. Interestingly, despite decreasing prognostic risks by PRKACA, it moderately expressed in HCC tissue, whereas underexpressed in normal tissues. The possible reasons were tumor heterogeneity and compensatory regulation.

### WNK1 Stimulates the Proliferation, Migration, and Invasion of Hepatoma Cells

**FIGURE 9:**
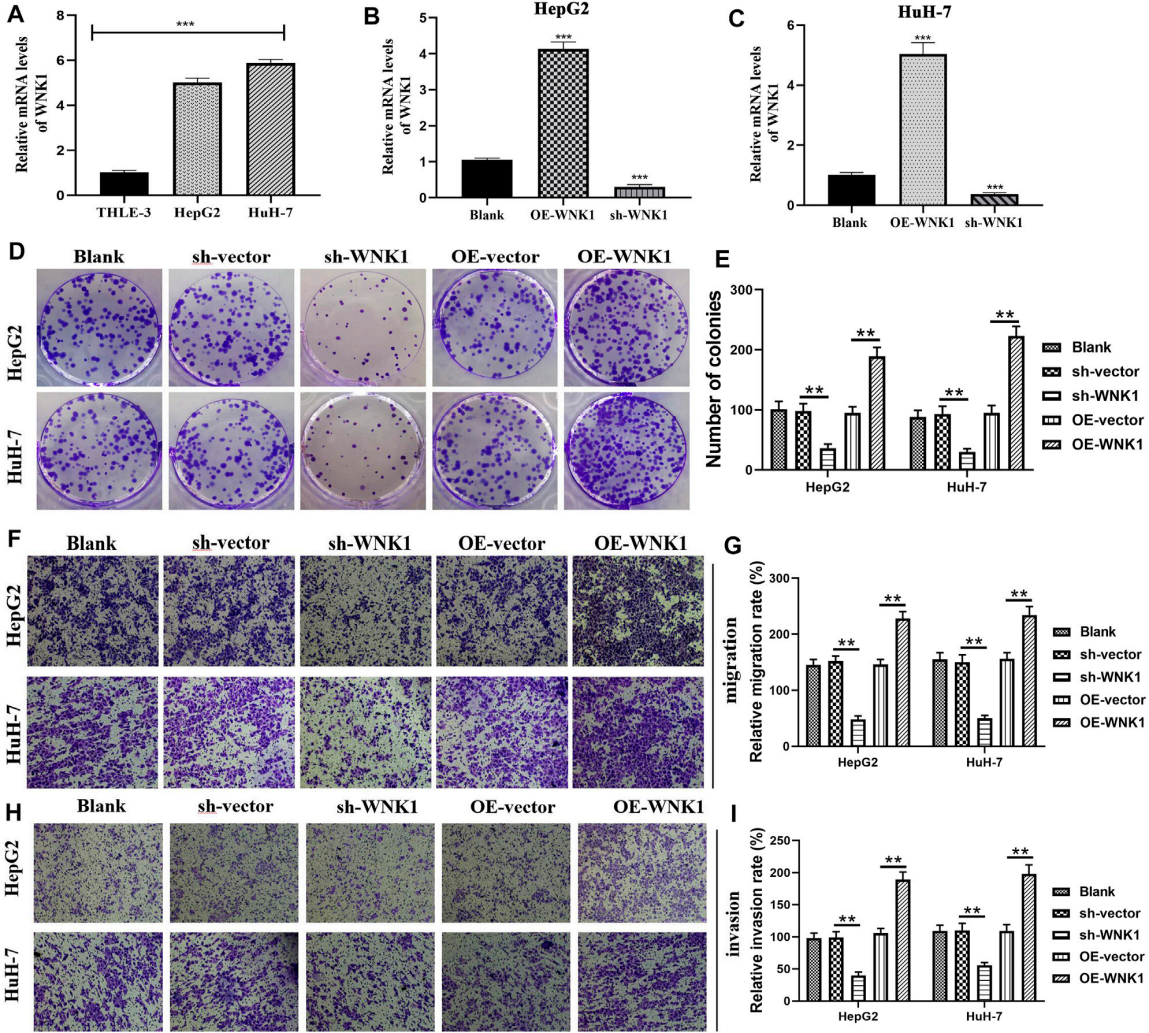
WNK1 promotes the malignant behaviors of hepatoma cells. **(A)** The expressive differences of WNK1 between normal liver epithelial (THLE-3) and hepatoma cells (HepG2 and Huh-7). **(B–C)** sh-WNK1 and OE-WNK1 effectively alter the mRNA expressions of WNK1. **(D–E)** WNK1 is proven to promote the proliferation of hepatoma cells through colony formation assays. **(F–G)** Transwell migration assays reveal that WNK1 enhances the migratory abilities of hepatoma cells. **(H–I)** Transwell invasion assays reveal that WNK1 enhances the invasive abilities of hepatoma cells. shRNA, short hairpin RNA; OE, overexpression plasmid; *p < 0.05, **p < 0.01, ***p < 0.001.

WNK1 is essential for angiogenesis and is intimately linked to some oncogenic signaling pathways, such as the TGF-β and PI3K-AKT pathways (Kankanamalage et al., 2018). In light of these facts, WNK1 attracts the attention of oncologists, which is the original intention of our experiments *in vitro*. WNK1 was significantly overexpressed in hepatoma cells (HepG2 and HuH-7) compared to normal liver epithelial cells (THLE-3) (Figure 9A). Specific shRNA (sh-WNK1) and overexpression plasmid (OE-WNK1) were confirmed to effectively alter WNK1 expression (Figure 9B,C). Colony formation assays revealed that the overexpression of WNK1 promoted the proliferation of hepatoma cells, whereas silencing WNK1 inhibited the proliferation of hepatoma cells (Figure 9D,E). Analogously, the overexpression of WNK1 enhanced migration of hepatoma cells, whereas blocking WNK1 expression suppressed the migration of hepatoma cells (Figure 9F,G). The same tendency was observed in cellular invasion ability (Figure 9H,I), WNK1 had a stimulative effect on the invasion of hepatoma cells. Collectively, WNK1 possessed tumorigenicity in HCC.

## Discussion

HCC is a common abdominal tumor with an incidence rate of 6–10 per 100,000 (Siegel et al., 2021). Owing to the low proportion of resectable cases and the limited efficacy of molecular target therapy (MTT) and immune checkpoint inhibitors (ICIs), its OS is commonly less than 40 months (Yang et al., 2019). Recently, pyroptosis demonstrated a promising capacity to fight cancers, which is the original intention of this study.

Due to the highly malignant tendency and invasive feature of HCC, accurate prognostic assessment is strikingly challenging. Regrettably, Beumer B.R’s team have proven that the current prognostic system, including the Barcelona Clinic Liver Cancer (BCLC) system, the American Joint Committee on Cancer (AJCC), the Cancer of the Liver Italian (CLIP) Program, and the Japan Integrated Staging (JIS) score, all performed poor in an external cohort (Beumer et al., 2021). Moreover, the c-index for the AJCC eighth edition system is only 0.60 (Kamarajah et al., 2018). Thus, developing a reliable prognostic system is crucial. In the current study, we constructed a novel PR risk model which elevated the decision-making benefit of the AJCC system (Figure 3F). Not only that, the PR risk score was identified as the only independent prognostic factor of HCC (Figure 3E) and had the ability to distinguish the survival difference of early-stage patients (T1/2 stage, Figure 3I). Given that either AJCC seventh edition or the latest eighth edition could not discriminate the prognostic difference in early-stage HCC (Satala et al., 2021), our PR risk score compensated for these weaknesses. Despite the lack of validation through a real clinical cohort, the prognostic value of the novel PR model was successfully verified in two external cohorts (n = 442). Compared with other types of models (Li et al., 2020; Zhen et al., 2021), our validation cohorts provided a larger sample size, which undoubtedly increased the model credibility. It is not difficult to perceive that our PR risk signature could act as an important supplement to the AJCC system.

It is well known that tumor size and clinical stage are pivotal prognostic markers in HCC. For example, Liang *et al.* have reported that in cirrhotic patients with solitary HCC and without macrovascular invasion, tumor size may significantly affect the prognosis after curative hepatectomy (Liang et al., 2021). However, the PR risk score was not related to tumor size and clinical stage in the GSE14520 cohort. The possible reason for this contradiction is tumor heterogeneity. As shown in table 1, the proportion of young patients (age<60) in the GSE14520 cohort (80.5%) was much higher than that in the TCGA (45.6%) and ICGC (19.0%) cohorts. Commonly, young HCC patients had high tumor malignancy and tend to suffer a poor prognosis (Kim et al., 2006), which may be the source of the heterogeneity among different cohorts. In contrast, we observed that PR risk score was positively associated with the advanced clinical stage (Supplementary Figure S1).

HCC is characterized by high immunogenicity. Its complex tumor immune microenvironment (TIM) directly determines the trend of antitumor immune and the efficacy of immunotherapy (Ringelhan et al., 2018). In the present study, high PR risk markedly decreased the infiltration level of CD8^+^ T cells and suppressed the activities of cytolytic function (Figure 5A,B). These alterations were all favorable to HCC progression. Existing studies have shown that pyroptosis plays a dual role in cancer regulation (Xia et al., 2019). On the one hand, as a type of PCD, pyroptosis itself is one of the approaches by which immune cells eradicate tumor cells. On the other hand, some inflammatory factors (IL-1β, IL18, and HMGB1) released by pyroptosis can trigger the activation of cancer-promoting signaling pathways including ERK1/2, p38 MAPK, and VEGF pathways (Lu et al., 2021). Interestingly, we found that the effects of PRGs on TIM also inherited this characteristic. For instance, moderately activated antigen-presenting cells (APCs) could elicit powerful immune responses against tumor cells (Sadeghzadeh et al., 2020). Reciprocally, excessive APC stimulation could drive the immune tolerance in TIM by upregulating the PD-1 and CTLA4 expressions in T cells (Wculek et al., 2020). Another example was type-II IFN response. High PR risk enhanced the activity of type-II IFN response (Figure 5B). Considering that type-II IFN commonly possesses antiproliferative, pro-apoptotic, and antitumor competencies (Castro et al., 2018), this change seems to contribute to the antitumor process. Nevertheless, type-II IFN could also induce immune evasion by downregulating MHC expression and upregulating PD-1/L1 expression (Castro et al., 2018). In a word, PRGs have complex and profound influences on the immune microenvironment of HCC.

Currently, nivolumab (PD-1 inhibitor), pembrolizumab (PD-1 inhibitor), and atezolizumab (PD-L1 inhibitor) have been recommended as first-line treatments for HCC patients by the latest NCCN guideline (Benson et al., 2021). Nonetheless, only a small fraction of patients can benefit from PD-1/L1 inhibitors. The available evidence indicates that PD-1/L1 can control GSDMC-mediated pyroptosis in cancer cells (Hou et al., 2020). Then, could pyroptosis in turn affect the expressions of ICs and the efficacy of immunotherapy? In the present study, we found that the PR risk score was positively correlated with the expressions of ICs (Figures 6A–G). Moreover, high PR risk resulted in more remarkable dysfunction of T cells and immune exclusion, which in turn rendered a higher TIDE score (Figure 6H). Hence, a high-PR risk score may serve as a biomarker for poor efficacy of ICIs. Sorafenib is another mainstream option recommended by the NCCN guideline for the systemic treatment of HCC (Benson et al., 2021). Similar to ICIs, the therapeutic response of sorafenib was also associated with the PR risk score. Hage C *et al.* have found that sorafenib could trigger NK cell-mediated cytotoxicity to combat HCC by inducing pyroptosis (Hage et al., 2019), which may be the theoretical basis for this therapeutic correlation. Moreover, according to the associations of PR signature genes with drug IC50 (Fig 7BCD), gefitinib, dasatinib, docetaxel, and 5-fluorouracil may be the sensitive drugs for HCC treatment.

WNK1 has been a research hot spot in the oncology field. Increasing evidence indicates the roles of WNK kinases which are represented by WNK1 in tumorigenesis by stimulating tumor cell proliferation (Sie et al., 2020). Therefore, the inhibition of WNK kinases may be a potent anticancer therapy. Besides, in 2021, Mayes-Hopfinger, L *et al.* have reported that WNK1 is capable of retarding pyroptosis through negatively regulating NLRP3(Mayes-Hopfinger et al., 2021), which witnesses that WNK1 acts as a crucial regulator in the pyroptotic process. Unfortunately, the biofunctions of WNK1 in HCC remain elusive. In contrast, NOD2 has been proven to promote hepatocarcinogenesis *via* a RIP2-mediated pro-inflammatory response (Zhou et al., 2021). Through further experiments *in vitro*, we have demonstrated that the overexpression of WNK1 could promote the proliferative, migratory, and invasive abilities of hepatoma cells, which unveiled its potent anticancer potential.

Naturally, there are some limitations that need to be noted in this study. First, the PR risk signature has not been confirmed in a real clinical cohort. Second, the assumption regarding the linkage between the PR risk score and the efficacy of PD-1/L1 inhibitors needs further validation. Third, we did not verify the biofunctions of all seven risk PRGs. Fourth, the oncogenic mechanisms of WNK1 were not deeply delved into. The resolution of these limitations awaits further research.

## Conclusion

HCC is a common and challenging tumor that exerts a great health burden on patients. In recent years, pyroptosis brings a promising paradigm shift to cancer treatment. In view of this, we elaborated on the roles of PRGs in the prognosis, TIM, and therapeutic response of HCC. Through Lasso regression analysis, a novel PR risk signature was constructed. The PR risk score was identified as an independent prognostic factor of HCC and extremely lifted the predictive performance of the TNM staging system. Moreover, high PR risk was detrimental to antitumor immune through changing the infiltrating levels of CD8^+^ T cells and macrophages. As for therapeutic correlation, the PR risk score may serve as a biomarker for predicting the efficacy of PD-1/L1 inhibitors and sorafenib. Given that WNK1 has recently received wide and eager attention from oncologists, its biofunctions were further confirmed by experiments *in vitro*. In conclusion, these findings provide new insights into the prognostic assessment and treatment of HCC.

## Data Availability

The original contributions presented in the study are included in the article/Supplementary Material, further inquiries can be directed to the corresponding author.
