# Supplementary material for: Pyroptosis-Related Risk Signature Exhibits Distinct Prognostic, Immune, and Therapeutic Landscapes in Hepatocellular Carcinoma
Source: Front Genet. 2022 Mar 9;13:823443. doi: 10.3389/fgene.2022.823443 (PMC8965507; doi:10.3389/fgene.2022.823443)
Supplement: Supplementary file 1 [file DataSheet1.ZIP › Data/Description.docx]

Pyroptosis-related----FR

Hepatocellular carcinoma----HCC

Differential expressed genes----DEGs

single-sample gene set enrichment analysis----ssGSEA

1. The improved PR gene set (n=45).

2. The expressions of 45 PR genes in TCGA database (n=424).

3. The expressions of 45 PR genes in normal liver tissues based on GTEx database (n=110).

4. The identification of PR DEGs (n=22).

5.The clinical information of TCGA cohort (n=377).

6. The PR risk score of each HCC sample in TCGA cohort (n=342).

7. The PR risk score of each HCC sample in GSE14520 cohort (n=221).

8. The PR risk score of each HCC sample in ICGC cohort (n=231).

9. The distribution of immune abundance of 22 leukocyte subtypes in each HCC sample (n=374).

10. The results of ssGSEA.

11. The immune score of each HCC sample based on ESTIMATE algorithm (n=374).

12. The expressive correlations between PR risk score and immune checkpoints (n=342).

13. The clinical information of IMvigor 210 cohort (n=348).

14. The relationships between PR risk score and the efficacy of sorafenib based on GSE109211 dataset (n=67).

15. The TIDE score of each HCC sample in TCGA dataset (n=424).

16. The effects of core PR risk genes on the sensitivity of multiple drugs based on GDSC database (n=265).

17. The clinical information of HPA database.
