# Supplementary material for: Pyroptosis-Related Risk Signature Exhibits Distinct Prognostic, Immune, and Therapeutic Landscapes in Hepatocellular Carcinoma
Source: Front Genet. 2022 Mar 9;13:823443. doi: 10.3389/fgene.2022.823443 (PMC8965507; doi:10.3389/fgene.2022.823443)
Supplement: Supplementary file 2 [file DataSheet2.ZIP › Supplementary Files/Supplementary table 2.docx]

Supplementary table 2. The clinical information of IMvigor210 and GSE109211 cohorts.

| Items | IMvigor210 | GSE109211 |
| --- | --- | --- |
| Publication Year | 2017 | 2018 |
| Country | the United States | Spain |
| Study (PMID) | 27939400 | 30108162 |
| Sample size | 348 | 140 |
| Tumor | mUC | HCC |
| Treatment | Atezolizumab | Sorafenib |

mUC, metastatic urothelial carcinoma; HCC, hepatocellular carcinoma.
