# Supplementary material for: Pyroptosis-Related Risk Signature Exhibits Distinct Prognostic, Immune, and Therapeutic Landscapes in Hepatocellular Carcinoma
Source: Front Genet. 2022 Mar 9;13:823443. doi: 10.3389/fgene.2022.823443 (PMC8965507; doi:10.3389/fgene.2022.823443)
Supplement: Supplementary file 2 [file DataSheet2.ZIP › Supplementary Files/Supplementary table 3.docx]

Supplementary Table 3. The specific sequences of sh-WNK1 and OE-WNK1

| Gene | Sequence (5' -> 3') |
| --- | --- |
| sh-WNK1 | CCGGCCGCGATCTTAAATGTGACAACTCGAGTTGTCACATTTAAGAT CGCGGTTTTTG |
| OE-WNK1 | WNK1-XbaI-F: **GCTCTAGA**ATGTCTGGCGGCGCCGCAGAGAAGCAG |
|  | WNK1-EcoRI-R: **GGAATTC**CTAAGTGGTCCGCAGGTTGGAGCCTGG. |

OE, over expression.
