# Supplementary figures and images for: Pyroptosis-Related Risk Signature Exhibits Distinct Prognostic, Immune, and Therapeutic Landscapes in Hepatocellular Carcinoma
Source: Front Genet. 2022 Mar 9;13:823443. doi: 10.3389/fgene.2022.823443 (PMC8965507; doi:10.3389/fgene.2022.823443)

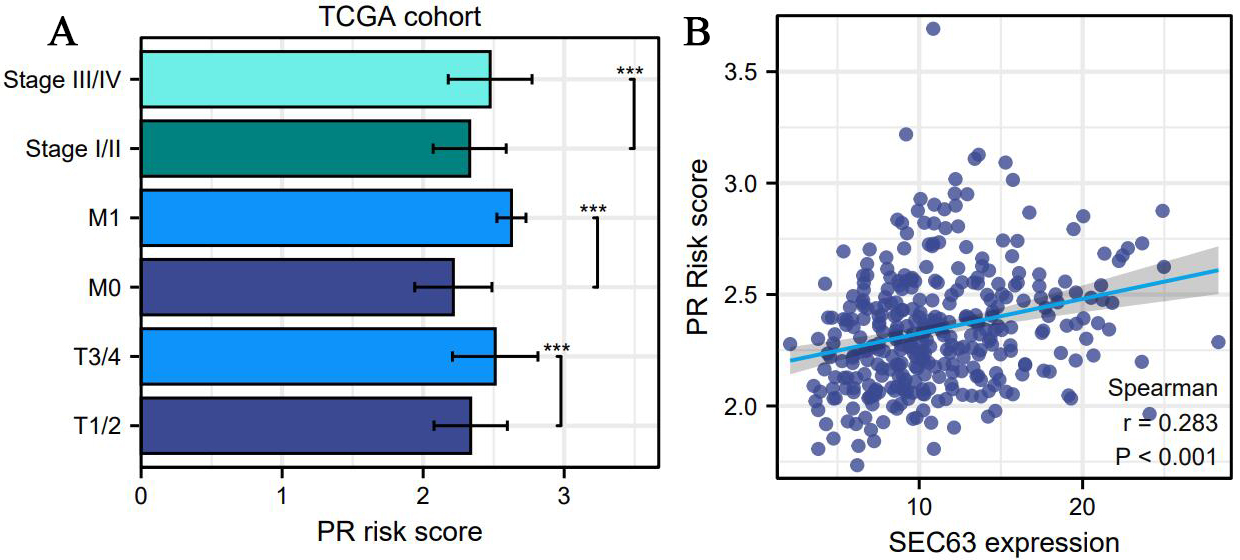

Supplement: Supplementary file 2 [file DataSheet2.ZIP › Supplementary Files/Supplementary figure 1.jpg]

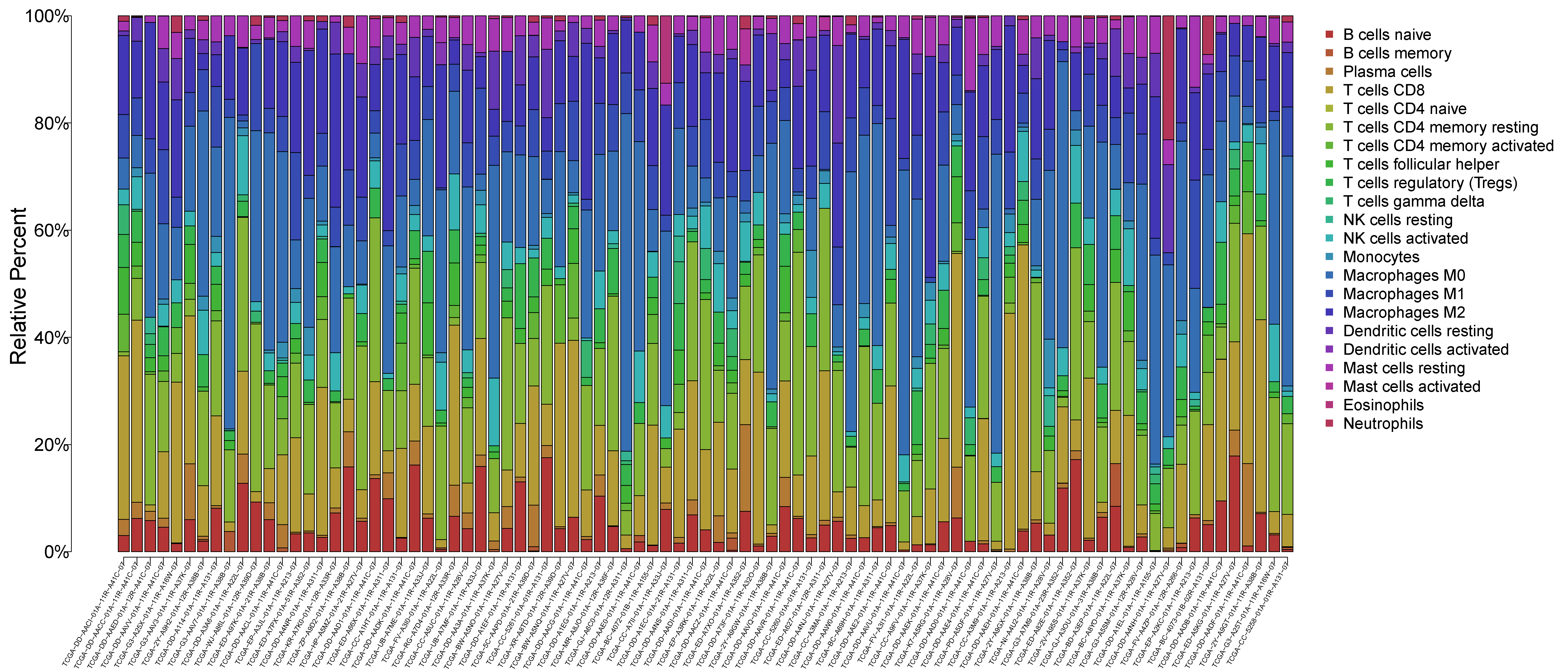

Supplement: Supplementary file 2 [file DataSheet2.ZIP › Supplementary Files/Supplementary figure 2.jpg]

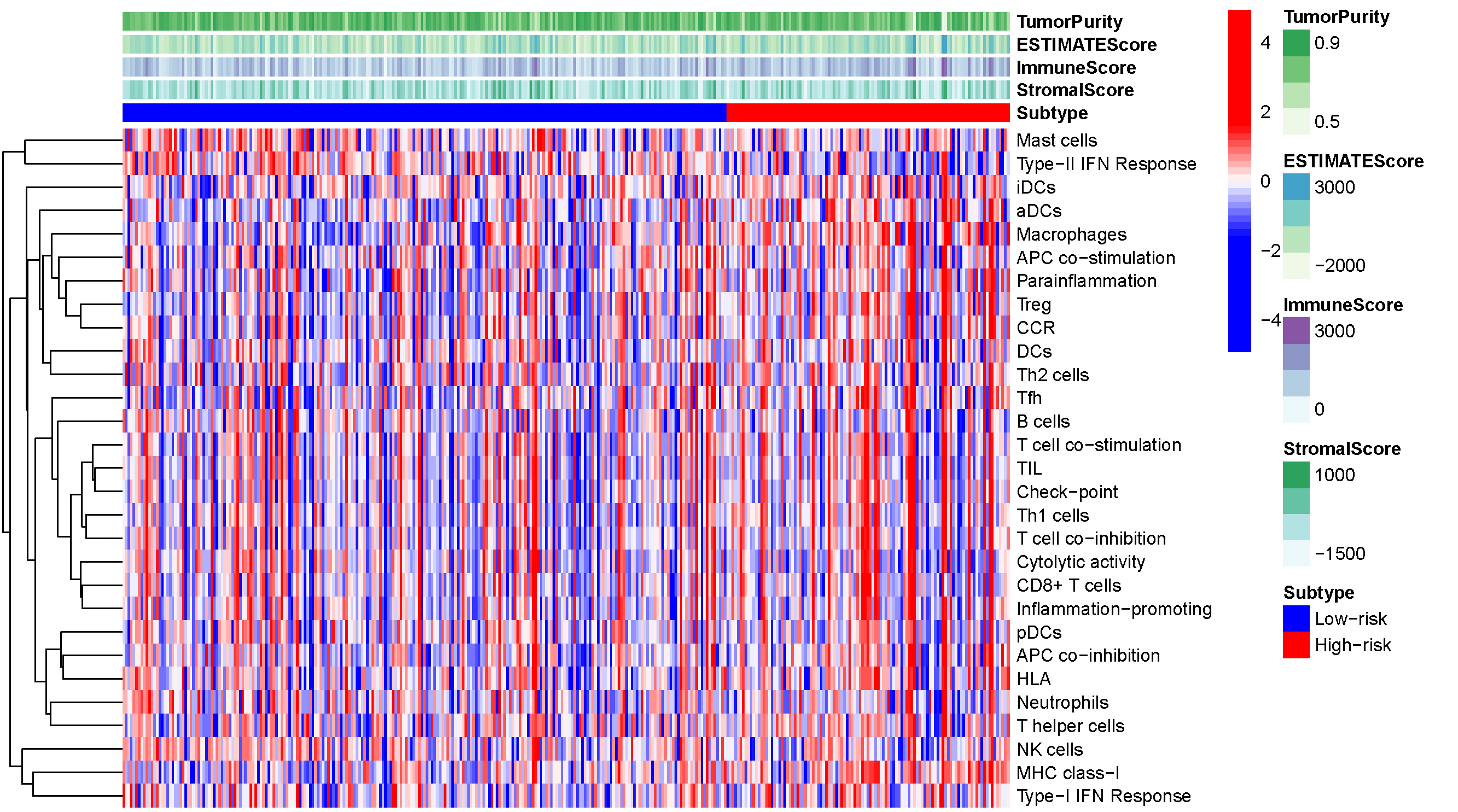

Supplement: Supplementary file 2 [file DataSheet2.ZIP › Supplementary Files/Supplementary figure 3.jpg]
